# White matter lesion filling improves the accuracy of cortical thickness measurements in multiple sclerosis patients: a longitudinal study

**DOI:** 10.1186/1471-2202-15-106

**Published:** 2014-09-08

**Authors:** Stefano Magon, Laura Gaetano, M Mallar Chakravarty, Jason P Lerch, Yvonne Naegelin, Christoph Stippich, Ludwig Kappos, Ernst-Wilhelm Radue, Till Sprenger

**Affiliations:** Department of Neurology, University Hospital Basel, Petersgraben 4, 4031 Basel, Switzerland; Kimel Family Translational Imaging Genetics Laboratory, Research Imaging Centre, Centre for Addiction and Mental Health, Toronto, Canada; Department of Psychiatry and the Institute of Biomaterials and Biomedical Engineering, University of Toronto, Toronto, Canada; Program in Neuroscience and Mental Health, The Hospital for Sick Children, Toronto, Canada; Department of Medical Biophysics, University of Toronto, Toronto, Canada; Department of Radiology and Nuclear Medicine, Division of Diagnostic and Interventional Neuroradiology, University Hospital Basel, Basel, Switzerland; Medical Image Analysis Center, University Hospital Basel, Basel, Switzerland

**Keywords:** Multiple sclerosis, Cortical thickness, Lesion filling, Longitudinal analysis

## Abstract

**Background:**

Previous studies have demonstrated that white matter (WM) lesions bias automated brain tissue classifications and cerebral volume measurements. However, filling WM lesions using the intensity of neighbouring normal-appearing WM has been shown to increase the accuracy of automated volume measurements in the brain. In the present study, we investigate the influence of WM lesions on cortical thickness (CTh) measures and assessed the impact of lesion filling on both cross-sectional/longitudinal and global/regional measurements of CTh in multiple sclerosis (MS) patients.

**Methods:**

Fifty MS patients were studied at baseline as well as after three and six years of follow-up. CTh was estimated using a fully automated pipeline (CIVET) on T1-weighted magnetic resonance images data acquired at 1.5 Tesla without (original) and with WM lesion filling (filled). WM lesions were semi-automatically segmented and then filled with the mean intensity of the neighbouring voxels. For both original and filled T1 images we investigated and compared the main CIVET’s steps: tissue classification, surfaces generation and CTh measurement.

**Results:**

On the original T1 images, the majority of WM lesion volume (72%) was wrongly classified as gray matter (GM). After lesion filling the accuracy of WM lesions classification improved significantly (p < 0.001, 94% of WM lesion volume correctly classified) as well as the WM surface generation (p < 0.0001). The mean CTh computed on the original T1 images, overall time points, was significantly thinner (p < 0.001) compared the CTh estimated on the filled T1 images. The vertex-wise longitudinal analysis performed on the filled T1 images showed an increased number of vertices in the fronto-temporal region with a significantly decrease of CTh over time compared the analysis performed on the original images.

**Conclusion:**

These results indicate that WM lesions bias the CTh estimation both cross-sectionally as well as longitudinally. The lesion filling approach significantly improved the accuracy of the regional CTh estimation and has an impact also on the global estimation of CTh.

## Background

Accurate brain tissue classification approaches are crucial for extracting useful information and developing reliable measures to describe brain morphological changes related to development and disease. Various sources of variability and inaccuracy may bias the computation of brain measurements based on magnetic resonance (MR) data. The quality of MR data (e.g. intensity inhomogeneity or partial volume averaging due to low resolution), differences of mathematical algorithms and brain tissue alterations due to pathologies may contribute to reduce the accuracy of brain tissue classification [1–4]. In this regard, the influence of the white matter (WM) lesions as observed in multiple sclerosis (MS) patients has been previously investigated. Indeed, on T1- weighted (T1w) images (MR sequences used in typical clinical neuro-scientific research settings), WM lesions are characterized by MR signal intensities close to gray matter (GM) and cerebrospinal fluid (CSF) introducing a possible bias on tissue classification. The outputs of typical classification algorithms that do not account for lesions may subsequently categorize these lesions as GM or, in some cases, as CSF. Previous works have demonstrated how lesion misclassification biases overall brain tissue segmentation [1, 5–7], leading to an overestimation of GM atrophy [6]. Various methods have been proposed to account for WM lesions in order to optimize tissue segmentation. Chard and colleagues [5], for instance, developed an automated method to fill the WM lesions with values approximating normal-appearing white matter (NAWM). They showed that GM and WM volumes were substantially affected by the misclassified WM lesions and that a lesion filling approach could reduce the classification error. Interestingly, Sdika and Pelletier [7] argued that, not only the segmentation, but also the image registration step could be affected by WM lesions. For this reason, they tested three different lesion filling methods: 1) they filled the lesions from their border to their center with an average of neighbouring voxels; 2) using only the value of the surrounding NAWM; 3) and using the mean intensity of the NAWM over the whole brain. They found out that the second approach led to optimal results in case of non-linear registration. Furthermore, in a recent paper, Battaglini and colleagues [1] compared two different methods to reduce the impact of WM lesions. One simply masked out the lesions from the original MR images, while the other refilled each lesion with intensities derived from a histogram of the WM surrounding the lesion. The latter approach significantly improved the accuracy of the tissue classification and brain volume measurements computed by SIENAX [8].

The tissue classification is a fundamental step not only for measuring volumes, but also for assessing more complex features of brain morphology such as cortical thickness (CTh). It has been shown that CTh can be reliably measured both globally as well as locally in healthy subjects and in patients with neurological and psychiatric disorders [9–11]. The currently used automated techniques [12, 13] estimate CTh using three main analysis steps. First, each voxels of the brain 3D T1w MRI data are classified into GM, WM and CSF and include estimates of partial volume. Then WM and GM surfaces are generated by using a three-dimensional polygonal mesh, and, finally, the CTh is computed as the distance between the two surfaces at each node (vertex). The accuracy of CTh measurements is strongly related to the accurate reconstruction of the inner and outer-surfaces of the cortex, which are in turn influenced by the tissue classification. Hence, we hypothesized that WM lesions could affect the reliability of CTh estimation. Although the number of studies investigating CTh in MS patients is increasing [14–23], to our knowledge, only few of them considered the WM lesions during tissue classification and when reconstructing the surfaces [16, 17, 21]. Interestingly, all these studies evaluated the relationship between WM lesion volume and CTh, but only one study explored the influence of WM lesions on the estimation of CTh [21]. The authors showed an increased accuracy of CTh estimation near the WM lesions after lesion masking.

In the present work, we explored the effect of WM lesions on the estimation of CTh in a group of MS patients. WM lesions were filled with the intensity of the normal-appearing neighboring voxels and we assessed the accuracy of brain tissue classification, surface reconstruction and CTh estimation. Moreover, a vertex-wise longitudinal analysis was performed comparing the CTh estimated on the original T1w images and on the filled images.

## Patients and methods

### Subjects

Data of fifty patients with MS (35 women, mean age: 45.69 ± 10.75 years, range: 21-64 years; mean disease duration at baseline: 15.9 ± 9.2 years), taking part in a longitudinal cohort study on the genotypic-phenotypic characterization of MS recruited at a tertiary center (Department of Neurology, University Hospital Basel), were retrospectively analysed. All patients underwent a thorough medical and structured neurological examination with Expanded Disability Status Scale (http://www.neurostatus.net; Table 1). MRI data at baseline (BL), after 3 years (Y3) and after 6 years (Y6) of follow-up were assessed. Written informed consent was obtained from each patient after a detailed explanation of all procedures. The study was approved by the local ethics committee (Ethikkommission beider Basel, EKBB) and was conducted in concordance with the Declaration of Helsinki.

**Table 1:** **Clinical characteristics of multiple sclerosis patients**

	CIS	RRMS	SPMS	PPMS	EDSS median (range)
BL	1	37	8	4	3 (0-6.5)
Y3	0	36	10	4	3 (1-7)
Y6	0	36	10	4	3.5 (0-7.5)

CIS: Clinical Isolated Syndrome; RRMS: Relapsing-Remitting Multiple Sclerosis; SPMS: Secondary Progressive Multiple Sclerosis; PPMS: Primary Progressive Multiple Sclerosis EDSS = Expanded Disability Status Scale; BL: baseline; Y3: follow-up after 3 years; Y6: follow-up after 6 years.

### MRI protocol

Morphological analyses were performed on high-resolution three-dimensional T1w MPRAGE images acquired in sagittal plane (TR/TI/TE = 2080/1100/3.0 ms; α = 15°, 160 slices, isotropic resolution of 1 mm^3^). Additionally, a double spin echo proton density (PDw)/T2-weighted (T2w) sequence was acquired (TR/TE1/TE2 = 3980/14/108 ms; flip angle 180, 40 slices, 3 mm slice thickness without gap with an in-plane resolution of 1 mm × 1 mm). All MRI scans were performed on a 1.5 Tesla Magnetom Avanto MRI scanner (Siemens Medical Solutions, Erlangen, Germany).

### MRI pre-processing

#### Lesion segmentation

White matter lesions were segmented by trained experts according to the structured operating procedures used at our institution for the analysis of clinical phase II and phase III trials. In brief, lesions are first marked by putting a cursor into the lesion and then semi-automatically segmented using intensity thresholding with Amira 3.1.1 (Mercury Computer System Inc.). Manual adjustments are performed when necessary. The lesions are marked on PDw images, while the according slices of T2w images are displayed to confirm the lesion site and extent. All raters undergo a training period with consecutive reliability testing before working on any study. Reliability is retested in all raters at fixed intervals (once a year). This ensures a consistently high quality of lesion marking and segmentation. After lesion marking and segmentation, there is a final quality control step with verification of all segmentations by a radiologist. Then, the mean WM lesion volume across patients was computed for the whole brain and for each lobe as segmented by the “Automatic Nonlinear Image Matching and Anatomical Labeling” algorithm (ANIMAL; [24]).

#### Lesion filling procedure

**Figure 1:**
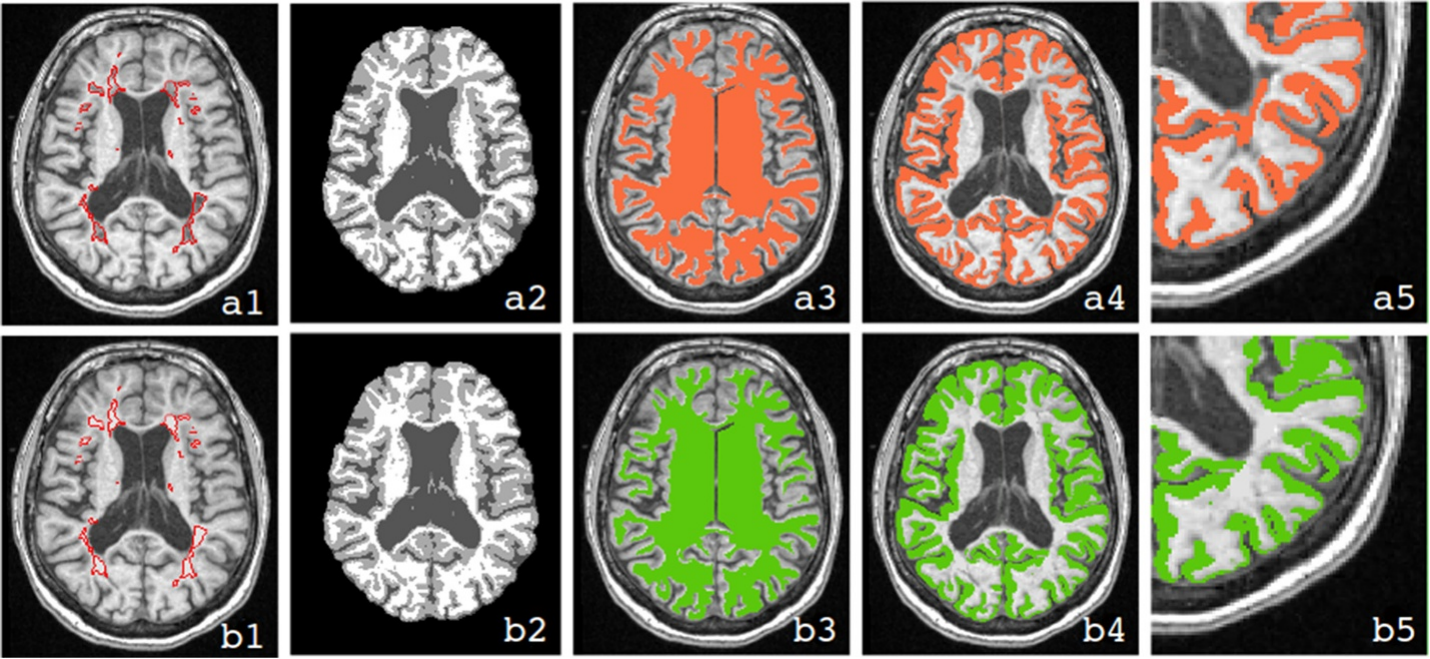
**Comparison of different analysis steps necessary for the measurement of the cortex with and without lesion filling.** The first row **(a)** illustrates the analysis strategy without lesion filling, while the second **(b)** illustrates the approach with lesion filling. In both rows, a T1w MRI **(a1 and b1)** with segmented lesions, the corresponding tissue classification derived from CIVET **(a2 and b2)**, the WM surface transformed back to volume space **(a3 and b3)**, the representation of the cortex **(a4 and b4)**, and a magnified view of the cortical thickness assessment **(a5 and b5)** are shown. The figure shows that the misclassification of WM lesions, which occurs using the approach without lesion filling **(a2)** produces an inaccurate WM surface **(a3)** and, consequently, an incorrect estimation of cortical thickness **(a4)** especially in the proximity of juxtacortical lesions **(a5)**. Using the approach without lesion filling, the estimated cortex in fact includes also lesional voxels.

The registration matrix between T1w and PDw images was estimated for each MRI session and applied to the binary lesion mask. In order to identify the neighbouring voxels of lesions that belong to WM, the registered and resampled lesion masks were expanded to the neighbouring two voxels in each direction. The lesion masks and the GM masks estimated on the original T1w images using the “Intensity Normalized Stereotaxic Environment for Classification of Tissues” algorithm (INSECT; [25]) were then subtracted from the expanded lesion masks. The mean voxel intensity was computed on the generated lesion border mask excluding voxels below the 10th percentile of signal intensity. The computed mean was used to fill the lesions on the original T1w images producing filled T1w images in the native space (Figure 1, a1 and b1). All steps were performed by using Medical Imaging NetCDF tools (MINC; http://en.wikibooks.org/wiki/MINC).

#### Cortical thickness

CTh was estimated on the T1w images (separately for original and filled images) by using the fully automated CIVET 1.1.10 pipeline [26, 27]. In brief, the images were linearly registered to the standard stereotaxic space defined by the MNI ICBM 152 model [28]. The images were then corrected for intensity non-uniformity using N3 [29] and a non-linear registration to the model [26] was applied. The tissue classification was performed using INSECT, whose output was then fed to a Partial Volume Estimator, which in turn is used for the actual surface fitting [30]. Each voxel was classified as WM, GM or CSF. The images were then mapped to a probabilistic atlas using the ANIMAL algorithm. Finally, the WM surface was generated by using a deformable ellipsoid polygonal model that shrinks until it fits the WM/GM interface. To generate the GM surface, the WM surface was expanded until the GM/CSF interface (or pial surface) is reached using a Laplacian approach in order to find the best fit [31, 32]. Specifically, to adequately estimate the CTh, the Laplace’s equation describes a smooth trajectory between the WM and GM surfaces defining a layered set of surfaces [32]. Thus, each vertex on the WM surface maps to a specific point in the GM surface and back to the same point in WM surface. The CTh is estimated as the distance, in millimetres, between WM and GM matter surfaces at each vertex. The surfaces are composed of 40,962 vertices for each hemisphere. Moreover, the mean CTh for each region-of-interest (ROI) generated by the ANIMAL algorithm was computed (for each hemisphere: frontal, parietal, occipital and temporal lobe, cingulate gyrus, splenium, parahippocampal gyrus and insula). Thickness data were blurred using a surface-based diffusion smoothing kernel of 20 mm full-width at half-maximum (FWHM) to be consistent with cortical topology [33].

### Data analysis

The mean WM lesion volume as well as mean juxtacortical WM lesion volume across subjects and sessions were computed separately. The results of the analysis strategies with and without lesion filling were compared at three critical steps: tissue classification, surface generation and CTh measurement. Regarding tissue classification, the overall percentages of voxels classified as WM, GM and CSF, and the mean voxel intensities for each segmented class were computed. The percentages of voxels (mis-)classified as WM, GM and CSF, but truly belonging to WM lesions were calculated as well.

**Figure 2:**
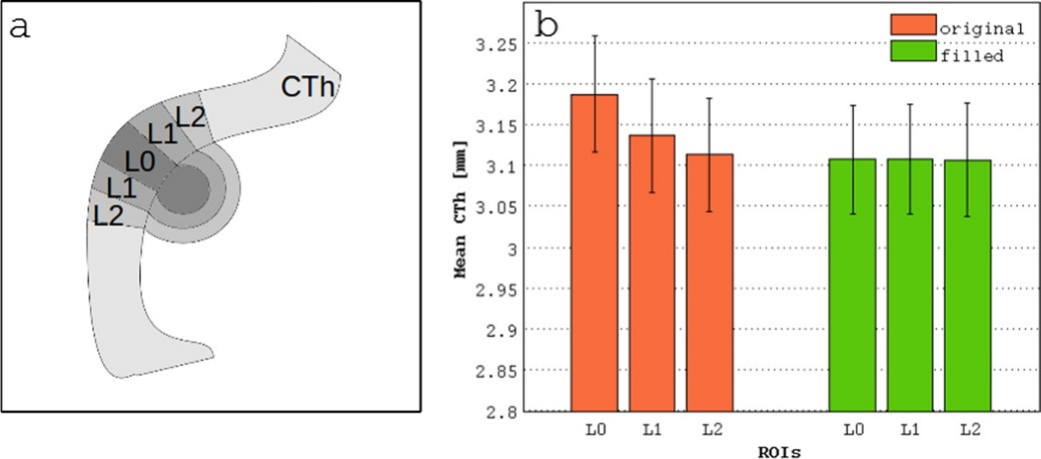
**Mean cortical thickness in proximity of juxtacortical lesions computed by the two analysis strategies over 6 years of follow-up in MS patients. a)** Three ROIs were drawn in order to evaluate how the approach with lesion filling performs in the presence of juxtacortical lesions: L0 represents the lesion and its border, L1 includes a two voxels rim surrounding L0 and may be consider as a transition zone between lesion and normal-appearing tissue. L2 includes the two voxels rim surrounding L1 and represents normal-appearing tissue. **b)** mean cortical thickness (CTh) and standard error for the defined ROIs. BL: baseline; Y3: follow-up after 3 years; Y6: follow-up after 6 years.

For the surface generation step, the WM and GM surface errors were automatically computed by CIVET separately for the approaches with and without lesion filling as the number of WM/GM voxels outside the WM/GM surface respectively (but inside the brain mask) divided by the number of voxels of the brain mask.Finally, for the whole brain and for each defined ROI the mean CTh was compared between the strategies with and without lesion filling for each time point. The local effect on CTh produced by the filling approach of regions next to juxtacortical lesions was investigated. In this regard, three regions of interest (ROI; Figure 2a) were built: L0 represented the lesion and its border, L1 included a two voxels rim surrounding L0, and L2 included a two voxels rim surrounding L1. The mean CTh in these three ROIs was computed as the average CTh at the vertices included in L0, L1 and L2. Longitudinal vertex-wise thickness changes derived from the two analysis strategies were also assessed.

### Statistical analyses

Normal-model based analysis of variance (ANOVA) was performed to investigate the differences between the approaches with and without lesion filling. Normality assumption was assessed by Shapiro-Wilk test [34] and homoscedasticity was assessed by the robust Brown-Forsythe version of the Levene’s test [35]. Non-parametric Wilcoxon signed rank test was used if data did not meet the assumptions of the linear model. The results (WM lesion classification, tissue volumes, tissues intensity and whole brain and ROI mean CTh) were compared both within each session and over the time points. In order to reduce the risk of type I errors the ROI results were corrected for multiple comparisons by using the False Discovery Rate (FDR) approach set at alpha levels of 0.05. Moreover, the vertex-wise longitudinal analysis was performed using a linear mixed model (http://www.bic.mni.mcgill.ca/ServicesSoftware/StatisticalAnalysesUsingR) including age at baseline, gender and time points as fixed-effects and patients as random-effect. All statistical analyses were performed using the R statistical environment (http://www.r-project.org).

## Results

The mean WM lesion volume across subjects was 11798 ± 7228 mm^3^ at BL, 12429 ± 7461 mm^3^ at Y3 and 12150 ± 7465 mm^3^ at Y6. The mean juxtacortical lesion volume was 5415 ± 5209 mm^3^ at BL, 6099 ± 5796 mm^3^ at Y3 and 5665 ± 5512 mm^3^ at Y6. Specifically, the frontal lobe was bilaterally the most affected region. The occipital lobe was less affected (see Table 2).

**Table 2:** **White matter lesion volume**

	Frontal		Parietal		Temporal		Occipital	
	Left	Right	Left	Right	Left	Right	Left	Right
BL	2742 ± 1943	2972 ± 2158	1729 ± 1322	1455 ± 1110	1307 ± 1021	1365 ± 1071	284 ± 349	185 ± 291
Y3	2938 ± 2024	2738 ± 1971	1808 ± 1339	1609 ± 1244	1464 ± 1183	1395 ± 1074	287 ± 362	200 ± 280
Y6	2873 ± 2037	2822 ± 2073	1815 ± 1420	1566 ± 1205	1352 ± 1116	1305 ± 1002	243 ± 309	174 ± 257

The table shows the mean and standard deviation of the WM lesion volume across subjects for each lobe. BL: baseline; Y3: follow-up after 3 years; Y6: follow-up after 6 years.

### Tissue classification

**Figure 3:**
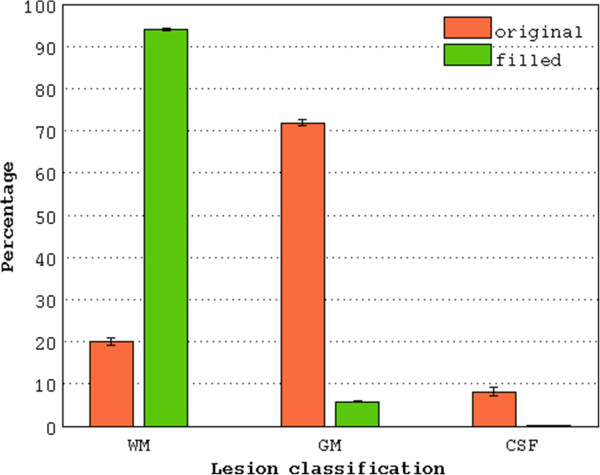
**Percentage of lesional voxels classified as WM, GM and CSF by the analysis strategies with and without lesion filling.** Using the original images without lesion filling, the majority of WM lesions are segmented as GM (71.90%). After lesion filling, the lesions are mostly classified as WM (94.02%), instead of GM or CSF (5.81% and 0.16% respectively ± standard error). WM: white matter; GM: gray matter; CSF: cerebrospinal fluid.

The WM lesions showed a lower mean signal intensity (Δ: 32 ± 2.4%) than the NAWM, i.e. non-lesional WM, on the original (non-filled) images at all time points, causing a misclassification of the majority of WM lesions as GM or CSF (Figure 3). The lesions correctly classified as WM, instead, were characterized by a mean intensity that was only 1.05 ± 1.28% lower than NAWM. The differences between the two analysis strategies in the ability to correctly classified WM lesion were tested performing a separated statistical model for each segmented tissue (GM, WM, CSF). The data showed a non-normal distribution (original: GM, W = 0.97, p < 0.001; WM, W = 0.93, p < 0.001; CSF, W = 0.81, p < 0.001; filled: GM, W = 0.91, p < 0.001; WM W = 0.9, p < 0.00; CSF, W = 0.52, p < 0.001) and violation of the assumption of homogeneity of variance (GM: F(1, 298) = 79.69, p < 0.001; WM: F(1, 298) = 69.89. p < 0.001; CSF: F(1, 298) = 115.4, p < 0.001). Thus, non-parametric analysis was performed. Significantly improved accuracy in classifying WM lesions as WM (V = 0, p < 0.0001, CI-95%: -77.8:-73.1) was observed after lesion filling (Figure 2). Accordingly, a significantly smaller volume of lesional WM was classified as GM (W = 11325, p < 0.0001, CI-95% = 64.7:67.8), and CSF (W = 11320, p < 0.0001, CI-95%: 5.1:8.1). Significant differences between the two analysis strategies at each time point were confirmed by pairwise analysis (p < 0.001, Bonferroni corrected). No significant differences of lesion classifications were observed between time points.

Using the approach with lesion filling, the mean intensity of voxels classified as WM and GM increased by 0.65 ± 1.7% and 0.3 ± 1.5%, respectively. On the other hand, the intensity of voxels classified as CSF decreased by 1.23 ± 7.5%. As a consequence, the overall time points analysis showed an increase of 1.45% of GM volume (mean difference between pipelines (ΔM): 12491 ± 63237 mm^3^, range: 152219: -248977), a decrease of 0.5% of WM volume (ΔM: 1043 ± 51347 mm^3^, range: 171981: -137716) and a decrease of 9.69% of CSF volume (ΔM: 15169 ± 29298 mm^3^, range: 139749: -71992) after lesion filling. The non-parametric analysis showed a statistically significant difference of GM volume between analysis strategies (W = 408, p < 0.02, CI-95% = -42311:-2429) at baseline, that did not survive for multiple comparisons correction based on Bonferroni approach. No other significant differences were observed.

### Surfaces

The WM surface generation errors were not normally distributed (original: W = 0.97, p < 0.01; filled: W = 0.96, p < 0.01) and violated the homogeneity of variance assumption (F(1, 298) = 16.52, p < 0.001). The performed non-parametric analysis showed a significantly improved accuracy of the WM surface generation after lesion filling (V = 11260, p < 0.0001, CI-95%: 1.2:1.6). The pairwise analyses showed significant differences between the two analysis strategies (p < 0.0001) for each time point. ANOVA testing did not evidence differences in GM surface generation errors between the analysis strategies (Table 3; Figure 1, a3 and b3).

**Table 3:** **Surface generation errors**

	Original		Filled	
	WM surface	GM surface	WM surface	GM surface
BL	12.62 ± 2.41 (8.66-20.56)	8.16 ± 1.37 (4.78-7.52)	11.16 ± 1.67 (8.49-16.49)	8.25 ± 1.28 (5.58-11.84)
Y3	12.74 ± 2.52 (8.21-17.81)	9.02 ± 1.55 (4.87-8.43)	11.16 ± 1.78 (8.17-16.54)	9.01 ± 1.44 (5.83-12.4)
Y6	13.28 ± 2.61 (8.65-20.24)	8.84 ± 1.52 (5.39-12.71)	11.7 ± 1.86 (8.4-16.45)	8.91 ± 1.41 (5.7-11.96)

The surface generation errors (mean ± standard deviation; min-max) computed as a percentage of classified WM/GM voxels outside the WM/GM surface respectively are reported for both analysis strategies (with and without lesion filling). WM: White Matter; GM: Gray Matter; BL: baseline; Y3: follow-up after 3 years; Y6: follow-up after 6 years.

### Cortical thickness

**Figure 4:**
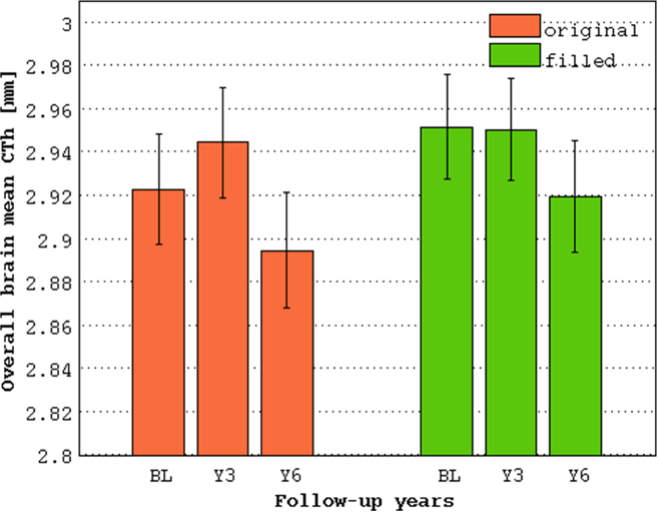
**Mean cortical thickness computed by the two analysis strategies over 6 years of follow-up in MS patients.** Using the data without lesion filling, an increase of the mean CTh after 3 years and a drop after 6 years is observed. Correcting the misclassification of WM lesions, instead, produces an evolution of the mean CTh as expected in MS patients. BL: baseline; Y3: follow-up after 3 years; Y6: follow-up after 6 years.

**Figure 5:**
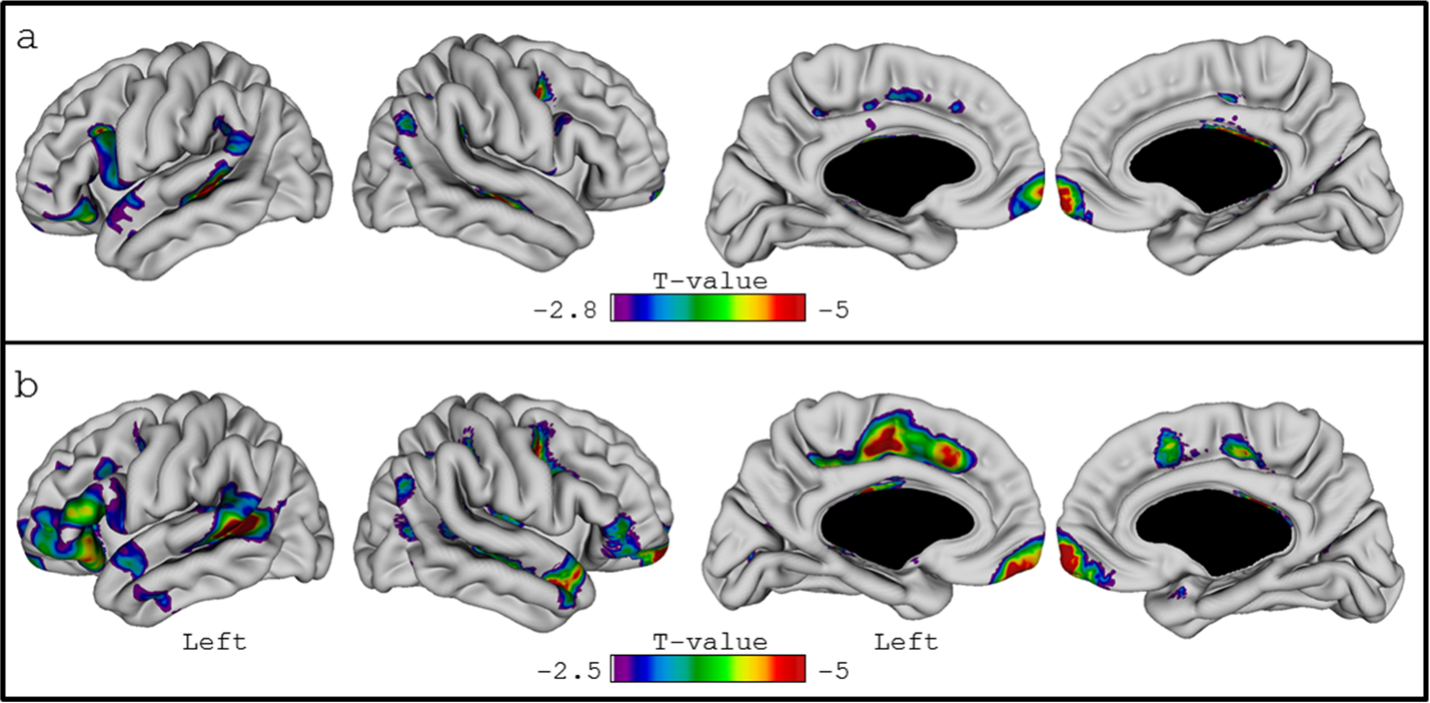
**Vertex-wise longitudinal analysis using the images with and without lesion filling.** The figure displays the results of the vertex-wise analysis performed on original (non-filled) **(a)** and filled **(b)** images using a linear mixed model including age (at baseline), sex, disease duration (at baseline) and time points as fixed effects and patients as random effect.

The mean CTh was not normally distributed (original: W = 0.97, p < 0.01; filled: W = 0.94, p < 0.001). The non-parametric analysis showed, over all time points, a significant thinner mean CTh measured on the original images compared to the filled ones (V = 3968, p < 0.001, CI-95%: -0.02:-0.005; Figure 4). The pairwise analyses showed significant differences between the two strategies (p < 0.0001) for each time point. After correction for multiple comparisons, bilateral differences were observed in the frontal lobe (left: V = 4555.5, uncorrected p (u.p) < 0.05; right: V = 4405, u.p < 0.01), in the parietal lobe (left: V = 4304, u.p <0.01; right: V = 4399.5, u.p < 0.05) and in the temporal lobe (left: V = 3938.5, u.p < 0.001; right: V = 3422, u.p < 0.0001). Moreover, differences in the left cingulate gyrus (V = 3788.5, u.p < 0.01), in the left splenium (V = 2727, u.p < 0.001) and left insula (V = 4250, u.p < 0.0001) were observed as well. At all time points, the local CTh measured in the three ROIs L0, L1 and L2 was not normally distributed (L0, original: W = 0.93, p < 0.0001; filled: W = 0.98, p < 0.05; L1, original: W = 0.96, p < 0.001; filled: W = 0.98, p = ns; L2, original: W = 0.98, p = ns; filled: W = 0.98, p < 0.05). The non-parametric analysis showed a significantly thicker cortex (Figure 2b) in the region close to the lesions compared to the neighbouring regions when using the original images (L0 vs L1: V = 8634, p < 0.0001; L0 vs L2: V = 8345, p < 0.0001). No significant differences of CTh between these regions were observed after lesion filling.The vertex-wise longitudinal analysis showed a significant CTh change over time in the frontal and temporal regions (Figure 5). The analysis performed on the filled images showed a more extended region of thinner cortex over time compared to the analysis performed on the original images (original: critical T value: -2.8, FDR = 0.05, vertices: 5395; filled: critical T value: -2.5, FDR = 0.05, vertices: 13389). Interestedly, after lesion filling, the standard error of the variable “years” decreased by 3.85% (on average, 0.0144 vs 0.0150) in the vertices close to juxtacortical lesions (i.e. belonging to L0).

## Discussion

In this work, we evaluated the influence of WM lesions on the estimation of CTh in patients with MS. The fully automated pipeline used in the present study was previously widely applied to investigate brain development [36], neurological [16, 17, 37, 38] and psychiatric [39] diseases.

Our main results support the view that lesion filling improves the accuracy of brain tissue classification, the generation of WM/GM surfaces and local CTh measurement. Based on these observations and taking into account the lack of an available ground truth we can only speculate that the increased accuracy observed in all preprocessing steps support the view that differences of global CTh measured between original and filled datasets reflect an increased accuracy of the measurement as well.

In line with the results reported in previous studies that investigated the influence of WM lesions on tissue classification [1, 5, 6, 40–42], we found smaller GM volumes using the original images compared with lesion filled images. The error in the assessment of GM volume can be partially explained by the darker WM intensity observed in the original images compared to the images with lesion filling. Accordingly, we observed that WM lesions accurately classified as WM had a lower T1w intensity than NAWM. This may cause a shift of the WM peak towards GM intensities in the image histogram, increasing the WM volume and decreasing the GM volume. In addition, a decreased mean intensity of CSF after lesion filling was observed. A higher intensity level of CSF may shift the CSF boundaries towards GM again reducing the GM volume.

On the other hand, it has been shown that if WM lesions are classified as GM, their effect on GM is in theory double: they directly increase the GM volume and they also cause a shift of GM boundaries towards higher intensity signal value, thereby decreasing the WM volume. In the non-filled images, the majority of WM lesions were classified as GM, but only the direct effect of increasing the GM volume was observed. In fact, the mean GM intensity did not change significantly after lesion filling, showing that the main effect is related to WM and CSF intensity changes.

The misclassification of GM/WM/CSF observed in the original images led to significant bias in the estimation of CTh as well. As described in the Patients and methods section, the tissue classification is one of the key steps in the analysis pipeline used in the present study to measure CTh. Specifically, after the classification of the voxels that belong to GM, WM and CSF, the WM and GM surfaces are extracted by using a deformable polygonal model [31] and the CTh is computed as the distance between WM and GM surfaces. We investigated the accuracy of the surface generation step on images with and without lesion filling, measuring the differences in percentage between the generated surfaces and the voxels classified as WM or GM outside the corresponding WM and GM surfaces. A significantly higher accuracy (i.e. a lower percentages of voxels not included in the surface) in the generation of WM surfaces after filling the lesions was observed. A reduced accuracy of the “Anatomic Segmentation using Proximity” (ASP; [43]) algorithm in the extraction of WM surface has been previously reported predominantly in the inferior portion of the brain. Because of the proximity of the ventricles, the algorithm has difficulties to stretch the surface sufficiently to match these regions [43]. Thus, it is possible that increased complexity of regional WM anatomy due to juxtacortical lesions classified as GM could reduce the accuracy of the “Constrained Laplacian ASP” (CLASP; [31]) algorithm that uses the same approach used by ASP to reconstruct the WM surface. We observed regional errors of the WM surface related to misclassified lesions in the images without lesion filling as shown in Figure 1 (a3). The surfaces are forced to include the lesions classified as GM producing a more convoluted CTh than it is in reality. This effect was completely eliminated using the lesion filling approach (Figure 1, b3).Including the lesions classified as GM (original images) in the computation of CTh (Figure 1, a5 and b5) caused the cortex to be significant thicker in the proximity of the lesions (i.e. L0) rather than the neighbouring regions (i.e. L1 and L2, Figure 2b), while in the filled images L0, L1 and L2 had similar CTh values. This means that when not accounting for lesions, misclassification can lead to focal changes in CTh values due to lesion characteristics and evolution rather than real changes of the CTh. Nonetheless, after lesion filling a very small percentage of voxels belonging to the WM lesion mask was still classified as GM. This could be related to slight errors in the lesion delineation, registration and/or resampling inaccuracy of the 2D lesion masks to the 3D T1w images and partial volume effects. Moreover, these potential sources of error could reduce the accuracy of the filling step. Indeed, portions of cortex could be wrongly classified as WM lesions (i.e. juxtacortical lesion) biasing the CTh estimation. We specifically investigated this issue comparing the CTh defined in the three ROIs next to juxtacortical lesions in the filled images (Figure 2b). As discussed before, we did not observe significant differences in CTh among L0, L1 and L2. Hence, we can conclude that the lesion segmentation, the lesion mask registration and resampling to the T1w images did not introduce significant errors.

In addition, the shifting of WM boundaries towards lower intensity values observed in the histogram of the images without lesion filling may produce a biased estimate of the CTh as well. In fact, the original images showed an overall thinner cortex compared to the mean CTh measured after the lesion filling at each of the assessed time point. Significant differences were observed in the frontal, parietal and temporal lobe bilaterally and in the left cingulate gyrus as well. This observation is consistent with the distribution of the white matter lesions.

Interestingly, the vertex-wise longitudinal analysis confirmed the relevance of using the lesion filling approach. A more extended fronto-temporal area of significant vertices was observed after lesion filling (Figure 5). Likely, the smaller cluster of significant CTh changes over time observed using the non-filled images relates to the variability between time points induced by the lesions. As previously demonstrated, CTh could be affected in a different way at each time point by the lesion changes of volume and intensity over time. Indeed, using the non-filled images, the mean CTh seemingly increased after three years and then decreased again after six years. As showed by the decreased standard error after lesion filling, the non-linear trend observed in the original images could reduce the goodness of fit of the linear-mixed model used to analyse the longitudinal data and consequently the statistical significance. A more linear decrease of CTh over the time points, instead, was observed after lesion filling. Moreover, we would like to highlight that in the longitudinal analysis the direction of the effect after lesion filling (increased vs. decreased of number of the significant vertices) could be specifically related to the dataset under evaluation. Indeed, the effect of lesions is related to lesion load and lesion distribution. Thus, it is not possible to judge the reliability of the reported results from previous studies that did not account for lesions without further analyses. However, according to our results, an increased variability between subjects is to be expected when not accounting for lesion, which should in turn reduce the statistical power. We would also like to underline that the method used in the present study to fill the WM lesions differs from other methods proposed in the past [1, 7]. The comparison between different WM lesion filling approaches was not the aim of the present study. Further studies that may compare different methods using the same MRI dataset are needed in order to identify the most accurate and robust lesion filling procedure.

## Conclusion

In the present study we have shown that WM lesions affect the estimation of CTh regionally by classifying lesions as part of cortex. The lesion filling approach significantly improved the accuracy of CTh estimation locally. Moreover, our results suggest that lesion filling has an impact also on the global estimation of CTh by shifting the WM/GM border.

## Abbreviations

ANIMAL: Automatic Nonlinear Image Matching and Anatomical Labeling
ANOVA: Analysis of variance
ASP: Anatomic Segmentation using Proximity
BL: Baseline
CSF: Cerebrospinal fluid
CLASP: Constrained Laplacian Anatomic Segmentation using Proximity
CTh: Cortical thickness
FDR: False Discovery Rate
GM: Gray matter
INSECT: Intensity Normalized Stereotaxic Environment for Classification of Tissues
MNI: Montreal Neurological Institute
MR: Magnetic resonance
MS: Multiple sclerosis
NAWM: Normal-appearing white matter
PDw: Proton density weighted
T1w: T1-weighted
T2w: T2-weighted
WM: White matter
Y3: Follow-up after 3 years
Y6: Follow-up after 6 years.
